# Formaldehyde in Dentistry

**Published:** 1899-04

**Authors:** George T. Baker

**Affiliations:** Boston, Mass.


### FORMALDEHYDE IN DENTISTRY.[1]

BY DR. GEORGE T. BAKER, BOSTON, MASS.

FORMALDEHYDE ($CH_2O$) is not a new product, but is one whose properties and uses are not at present fully understood. For a number of years it has been known that it was a most powerful germicide, antiseptic, disinfectant, and deodorant, and at the same

---

[1] Read before the American Academy of Dental Science, December 7, 1898.

time comparatively harmless to the higher forms of life. In addition to these qualities it is a very diffusible gas, and for this reason has been adopted by almost all the large cities for disinfection of dwellings and their contents, after cases of infectious diseases. It is also used in the preservation of foods. One part formaldehyde in thirty-two thousand is said to preserve milk for several days.

Gelatin when mixed with formaldehyde is insoluble in boiling water. This fact is useful in photography.

The fact that water absorbs the gas readily to the extent of a forty-per-cent. solution renders it easy of application as a disinfectant in the fluid form. It is this aqueous solution that is found in the market, and it can be mixed with water to form any degree of strength desired.

The different uses to which it has so far been put and the strength in which it seems best adapted to exert its antiseptic and hardening powers are given in a table by F. J. C. Bird (*Pharmaceutical Journal*). The one part of formaldehyde in this table represents two and one-half parts of the full strength, or forty-per cent. solution of commerce.

A SOLUTION OF FORMALDEHYDE.

1: 250,000 kills anthrax bacilli.

1: 50,000 prevents the development of typhus bacilli, etc.

1: 32,000 preserves milk for several days.

1: 25,000 forms a useful injection in leucorrhœa, etc.

1: 20,000 preserves wines, weak alcoholic liquids, and beer; also milk for several weeks.

1: 4000 recommended for moistening paper used to cover jam, etc.

1: 3200 for rinsing dairy vessels, etc.

1 : 2500 destroys the most resistant micro-organism in one hour.

1: 2000 for rinsing casks, and vessels intended for liquids liable to fermentation.

1: 500 as a mouth-wash.

1: 250 to 200 is a general disinfectant solution for washing hands, instruments, etc., in surgery, spraying in sick-rooms, as a deodorant.

1: 160 to 100 hardens microscopic tissues, which should be immersed for a considerable time to give the best results.

1: 100 in lupus, psoriasis, and skin-diseases.

1: 50 to 25 sterilizes surgical catgut, silk, etc., by steeping.

1: 25 for quickly hardening and preserving for microscopical sections; longer immersion in a weaker solution gives better results.

1: 10 for hardening very firm tissues in pathological and histological work.

1: 5 for hardening firm tissues in such work.

1: $2\frac{1}{2}$ for hardening soft tissues for the same purpose.

The fact that formaldehyde is non-poisonous and has no odor of its own but what is easily dissipated makes it useful in many different ways. We have so many products useful in dentistry as germicides, disinfectants, antiseptics, and deodorants, that it may seem unnecessary to add to the list, but formaldehyde seems to possess qualities that in some cases recommend it above all others.

For sterilizing instruments it seems to be of great value. It is efficient, neat, quick, and inexpensive. The instruments may be immersed in a solution of formaldehyde, but a far better way is to expose them to the gas.

Since the warm dry gas does not attack metal or injure in any manner the most delicate material, all sorts of instruments can be easily and quickly sterilized. This includes the hand-piece of the dental engine, rubber-dam holders, napkins, operating-coats, —in fact, everything connected with the office.

Drs. Reik and Watson, whose work is reported in the Johns Hopkins Hospital *Bulletin* (December, 1897), have experimented in this direction, and after giving their work in detail they state "that instruments can be sterilized in a small chamber in from ten to fifteen minutes, using respectively five or three grains of paraform to the cubic foot of space."

Some three months later (March, 1898) Dr. Reik made a supplementary report, in which he repeated his former statements and said, "My conclusions are, then, that we have in this method a rapid, cheap, easy, and sure method of sterilizing instruments without in any way injuring them."

To corroborate the above, we have the report of Elmer G. Horton, B.S., from the laboratory of Hygiene, University of Pennsylvania. (*Dental Cosmos*, July, 1898.) Mr. Horton took chisels, excavators, and burs, proved them sterile, infected them from cases in the operative clinic of the department of dentistry, placed them in a sterile tube, and dried them in an incubator for three hours.

Those infected instruments were then exposed to the fumes of formaldehyde for from ten to fifteen minutes.

After explaining the results in detail, Mr. Horton says, "We conclude that infected dental instruments can be disinfected without injury in a closed space of less than one cubic foot, by an exposure of fifteen minutes to the formaldehyde gas generated from a pastil containing five grains of paraform by heating the pastil over a proper alcohol lamp."

These and other experiments of men of authority all point to the fact that in formaldehyde we have a very desirable product and one that for many purposes in dentistry is bound to replace other inferior and objectionable disinfectants.

